# Immunotherapeutic efficacy of liposome-encapsulated refined allergen vaccines against *Dermatophagoides pteronyssinus* allergy

**DOI:** 10.1371/journal.pone.0188627

**Published:** 2017-11-28

**Authors:** Urai Chaisri, Anchalee Tungtrongchitr, Nitaya Indrawattana, Panisara Meechan, Watchara Phurttikul, Natt Tasaniyananda, Nawannaporn Saelim, Wanpen Chaicumpa, Nitat Sookrung

**Affiliations:** 1 Department of Tropical Pathology, Faculty of Tropical Medicine, Bangkok, Thailand; 2 Department of Parasitology, Faculty of Medicine Siriraj Hospital, Mahidol University, Bangkok, Thailand; 3 Center of Research Excellence on Therapeutic Proteins and Antibody Engineering, Faculty of Medicine Siriraj Hospital, Mahidol University, Bangkok, Thailand; 4 Department of Microbiology and Immunology, Faculty of Tropical Medicine, Bangkok, Thailand; 5 Department of Research and Development, Faculty of Medicine Siriraj Hospital, Mahidol University, Bangkok, Thailand; Chang Gung University, TAIWAN

## Abstract

Allergen specific immunotherapy (AIT) can modulate the allergic response causing a long-term symptom subsidence/abolishment which leads to reduced drug use and prevention of new sensitization. AIT of house dust mite allergy (HDM) using the mite crude extract (CE) as the therapeutic agent is not only less effective than the AIT for many other allergens, but also frequently causes adverse effects during the treatment course. In this study, mouse model of *Dermatophagoides pteronyssinus* (Dp) allergy was invented for testing therapeutic efficacies of intranasally administered liposome (L) encapsulated vaccines made of single Dp major allergens (L-Der p 1, L-Der p 2), combined allergens (L-Der p 1 and Der p 2), and crude Dp extract (L-CE). The allergen sparing intranasal route was chosen as it is known that the effective cells induced at the nasal-associated lymphoid tissue can exert their activities at the lower respiratory tissue due to the common mucosal traffic. Liposome was chosen as the vaccine delivery vehicle and adjuvant as the micelles could reduce toxicity of the entrapped cargo. The Dp-CE allergic mice received eight doses of individual vaccines/placebo on alternate days. All vaccine formulations caused reduction of the Th2 response of the Dp allergic mice. However, only the vaccines made of single refined allergens induced expressions of immunosuppressive cytokines (*TGF-β*, *IL-35* and/or *IL-10*) which are the imperative signatures of successful AIT. The data emphasize the superior therapeutic efficacy of single refined major allergen vaccines than the crude allergenic extract vaccine.

## Introduction

House dust mites (HDM), particularly *Dermatophagoides pteronyssinus* (Dp) and *D*. *farinae* (Df), produce a myriad of proteins and macromolecules that sensitize humans to allergic diseases including allergic dermatitis, allergic rhinitis and asthma worldwide [1]. Sensitization by the mites has been found in ~85% of asthmatic subjects [2]. Currently, 23 groups of the Dp- and Df- derived allergens have been recognized. Among them, group 1 (Der p 1 and Der f 1) and group 2 (Der p 2 and Der f 2) allergens are the most abundant and immunodorminant as more than 80% of the mite allergic subjects have serum IgE specific to them [3–6]. The group 1 allergens are highly cross-reactive in terms of their ability to induce T cell response and serum IgE binding [7]. Their allergenicity is attributable to their cysteine protease activity [8,9]. Der p 1 digests lung epithelium tight junctions (ZO-1) to increase accessibility of the allergen to the antigen presenting cells (APC) in the deeper tissue [10,11], cleaves low affinity IgE receptor (FcεRII or CD23) on human B cells and upset IgE regulation [12,13], cleaves α-subunit of human IL-12 receptor (CD25) on T cells leading to more IL-4 and less IFN-γ production [14], and digests α1-antitrypsin to promote airway inflammation and asthma [15]. Der p 1 also conditioned the airway dendritic cells (DCs) by digesting CD40 and DC-SIGN to diminish IL-12 and thiol secretion leading to a Th2 response bias to the allergen [16–18]. The HDM group 1 allergens cause release of pro-inflammatory cytokines from bronchial epithelial cells, tissue mast cells and blood basophils and increase allergen specific IgE production [19–22]. The biological functions of the HDM group 2 allergens (Der p 2 and Der f 2) are elusive. Der p 2 and Der f 2 have approximately 87% sequence identity and they share tertiary structure [23]. Their structure is similar also to the MD-2 (bacterial lipopolysaccharide binding protein) [24] and Niemann-Pick Type 2 (NPC2; cholesterol-binding protein) [25]. IgE epitopes of the group 2 allergens are more dependent on the conformational structure than the linear sequences [26].

Allergen specific immunotherapy (AIT) has curative potential for allergy. AIT not only mediates long-term mitigation or abolishment of the allergic symptoms which leads to reduction of drug usage, but also prevents sensitization to new allergen [7,27–31]. The mechanisms of AIT and immune tolerance to allergens have been reviewed recently [32]. It is evident that the AIT causes generation of regulatory T cells (Tregs) and B cells (Bregs) which produce immunosuppressive cytokines including IL-10, TGF-β and IL-35 [32–34]. Tregs and their cytokines suppress effector T cells and control the allergic diseases by several means which lead to reduced production of Th2 cytokines (IL-4, IL-5, IL-13); suppression of T cell homing to tissues; very early basophil tolerance and early decrease of mast cell and basophil activity; decrease numbers and mediator secretions of tissue mast cells and eosinophils; and suppression of excessive IgE synthesis and induction of IgG4 and IgA by allergen-specific B cells [32]. Bregs indirectly inhibit differentiation of Th1 and Th17 cells by suppressing pro-inflammatory cytokine production by DCs [35–37]. Also through production of TGF-β, Bregs induced apoptosis and anergy of effector CD4^+^ and CD8^+^ T cells, respectively [38,39]. Several studies have demonstrated the efficacy of the AIT for a variety of allergens including pollens, grass, trees, Hymenoptera sting, pet and HDM [40]. Nevertheless, the immunotherapy of HDM allergy by using crude mite extracts was not only less effective than the other allergen immunotherapies, but also frequently causes adverse effects, both local and systemic (anaphylaxis), especially in children [41]. The crude extracts may induce also new IgE reactivity to other mite components [42]. Thus, there have been attempts to reduce the side effect of the HDM immunotherapy by using either recombinant allergens, allergen-derived T cell peptides or recombinant hypoallergenic allergen derivatives prepared by different methods instead of the native crude HDM extract [43–49]. In this study, a fundamentally different approach was attempted for immunotherapy of the HDM allergy. Details of the experiments and results of the immunotherapy are reported herein.

## Materials and methods

### Preparation of whole body (crude) extract of *D*. *pteronyssinus* (Dp)

Live adult Dp collected from the cultures maintained at the Department of Parasitology, Faculty of Medicine Siriraj Hospital, Mahidol University, Bangkok, were washed with distilled water. Each gram of the cleaned mites were homogenized in 4 ml of phosphate buffered saline, pH 7.4 (PBS) by sonication (OmniRuptor 4000 Ultrasonic Homogenizer, OmniInternational, Georgia, USA) at 4°C for 15 min using power level 35 and pulse-off 50%. Protein content of the crude mite extract (Dp-CE) was determined using Bradford reagents (Bio-Rad, CA, USA).

### Preparation of Dp allergic mice

All animal experiments were approved by the Siriraj Animal Care and Use Committee, Faculty of Medicine Siriraj Hospital, Mahidol University (SiACUC no. 013/2558). For preparing Dp allergic mice, 6–8 week old male BALB/c mice (National Laboratory Animal Center, Mahidol University) were sensitized with Dp-CE as described previously [50]. Individual mice were injected intraperitoneally (i.p.) with three doses of 150 μg of Dp-CE in PBS mixed 1:3 (v/v) with alum adjuvant (Pierce, USA) on days 0, 7 and 14. On day 21, each mouse was challenged intranasally (i.n.) with 200 μg of the Dp-CE in 20 μl of normal saline solution (NSS) (10 μl into each nostril). On days 23, 25 and 27, the mice were nebulized with 10 ml of aerosolic PBS containing 10 mg of the Dp-CE. Sham mice were also prepared and they were injected i.p. with PBS mixed with alum, challenged i.n. with 20 μl of PBS and nebulized with 10 ml of aerosolic PBS using the same timeline as for the Dp-CE allergenized mice.

### Mouse sample collection

On day 28 (one day after nebulization), all mice were bled and the sera were collected separately for determining specific IgE and IgG1 by indirect ELISA [50]. After bleeding, four each of the allergenized, sham and naive mice were sacrificed by cervical dislocation performed by qualified veterinarian and/or scientist who hold certificates for use of laboratory animals in research from the National Research Council of Thailand (NRCT). Diaphragmatic lobe of the right lung of each mouse was preserved in 5% paraformaldehyde and 4% sucrose in PBS for 24 h. The fixed lung was embedded in paraffin and 5-μm sections were prepared. Sections were stained with hematoxylin and eosin (H & E) dyes and examined under a light microscope for histological features of the bronchioles and lung parenchyma. The remaining portion of the lung was preserved in an RNA stabilization reagent (RNA Later^™^, Qiagen GmbH, Germany) at -80°C. Total RNA was isolated from each preserved sample and used as a template in the study of cytokine expression profile by quantitative reverse transcription-PCR (qRT-PCR).

### Indirect ELISA

The indirect ELISA was used for determination of the mouse serum IgE and IgG1 specific to the Dp-CE. The assay was performed as described previously [50]. Each well of an ELISA plate was coated with Dp-CE (1 μg in 100 μl carbonate-bicarbonate buffer, pH 9.6) and blocked with 200 μl of 1% BSA in PBS. Triple Dp-CE-coated wells were added with individual mouse serum samples (diluted 1:4 for IgE and 1:100 for IgG1 determinations). After keeping the plates at 37°C for 1 h, all wells were washed with PBS containing 0.05% Tween-20 (PBST) and then added with 100 μl of rat anti-mouse IgE-biotin (Abcam^®^, Cambridge, USA) diluted 1:3,000 in PBST for IgE detection or goat anti-mouse IgG1-biotin (Southern Biotech, Birmingham, Alabama, USA) diluted 1:10,000 for IgG1 detection. The plates were incubated at 37°C for 1 h. Streptavidin-horseradish peroxidase conjugate (Southern Biotech) and freshly prepared 2,2’-azino-bis(3-ethyl benzothiazoline-6-sulfonic acid; ABTS) substrate (Gaithersburg, MD, USA) were used for color development. The color reaction was stopped by adding 50 μl of 1% SDS to each well. Wells added with PBS instead of the mouse serum served as blank. Optical density (OD) at 405 nm of the content in each well was determined against the blank. Data are presented as mean ± standard deviation (SD) of OD_405nm_ of serum samples of mice of the same group.

### Histologic study of the mouse lungs

H & E stained mouse lung sections were observed by a pathologist who was blinded to the mouse treatment groups. The degrees of the histopathological features in the bronchioles and lung parenchyma including the numbers of inflammatory cells infiltrated into peribronchiolar areas and morphology of the bronchiolar structure (epithelial cells and subepithelial layer) were evaluated using scoring system, 0–4, as described previously [50].

### Quantitative RT-PCR

The qRT-PCR was used for measuring cytokine gene expressions [50]. Total RNAs extracted from the lung tissues were treated with RNase-free DNase 1 (Invitrogen, CA, USA) and cDNAs were synthesized. The cDNAs were used as templates for quantification of mRNAs of cytokine gene expressions including *IL-4*, *IL-5*, *IL-6*, *IL-10*, *IL-13*, *IL-17A*, *TNF-α*, *IFN-γ*, *IL-12A (p35)*, *IL-12B (p40)*, *TGF-β*, and *IL-35* (*ebi3*). The nucleotide primers for amplification of the genes are listed in S1 Table. The qRT-PCR was performed on 1 μl of cDNA and 300 nM of each PCR primer in SYBR Green PCR Master Mix (Applied Biosystems, USA). A StepOne^™^ Real-time PCR system was run for 40 cycles and data were analyzed using StepOne^™^ software version 2.1. The expressions of individual genes were normalized to the mRNA of β-actin gene. Data are shown as fold change of the mRNA expression of the target gene in comparison to the β-actin mRNA level.

### Preparation of native (n) Der p 1 and nDer p 2

Native Der p 1 and nDer p 2 were prepared from the Dp-CE by using specific monoclonal antibody-based affinity resins as described previously [51]. Briefly, monoclonal IgG antibodies specific to Der p 1 or Der p 2 were fixed separately onto CNBr-Sepharose beads (GE Healthcare, Buckinghamshire, UK). After blocking the empty sites on the beads with 5% BSA in PBS, Dp-CE was added to mix with the affinity beads and the preparation was kept overnight on a rotating platform at 4°C. Unbound components were removed by washing with PBS and the affinity resin was packed into a 10 × 100 mm column. The resin was washed with PBS until the effluent had no detectable OD at 280 nm. The column bound protein was eluted from the affinity beads by using glycine-HCl buffer, pH 2.0, and immediately added with few drops of 1 M Tris-HCl, pH 8.5. The nDer p 1 and nDer p 2 preparations were dialyzed against distilled water and lyophilized. Protein contents of the preparation were determined (Bradford assay).

### Preparation of liposome

Multilamellar liposome was prepared as described previously [50]. Lipoid-S-100 phosphatidylcholine (PC) derived from fat-free soybean lecithin (Lipoid AG, Switzerland) and cholesterol (C) (Sigma-Aldrich, Germany) were used for preparing the micelles. Didecyldioctadecylammonium bromide (DDAB; Fluka, Germany) was used as a cationic surfactant. Lipid stock was prepared by mixing 153 mg DDAB, 148 mg PC and 72.5 mg C (molar ratio 2:1:1) in 25 ml dichloromethane. One ml aliquots of the lipid stock (30 μM) were rotated manually in round bottom flasks until a dried thin film was obtained in each flask.

### Liposome-encapsulated vaccines and the vaccine characterization

Four vaccine formulations were prepared: 1) liposome-encapsulated Dp-CE (L-CE); 2) liposome-encapsulated nDer p 1 (L-Der p 1); 3) liposome-encapsulated nDer p 2 (L-Der p 2); and 4) liposome-encapsulated mixture of the nDer p 1 and nDer p 2 (L-Com). Vaccine components, i.e., 5 mg of Dp-CE; 500 μg of either nDer p 1 or nDer p 2; or mixture of nDer p 1 and nDer p 2 (250 μg each) in 500 μl PBS were mixed separately with the gel film prepared from 1 ml of the lipid stock until a homogeneous creamy preparation was obtained. Placebo (L-PBS) was 500 μl PBS added to mix with the lipid film. Endotoxin contents in 500 μl of individual vaccine preparations were determined [Limulus amebocyte lysate (LAL) test kit; Associates of Cape Cod, Inc. (ACC), MA, USA] and amount of the endotoxin in each vaccine dose was calculated.

Zeta potential, polydispersity and sizes of the vaccines and placebo were determined by means of dynamic light scattering under an automatic mode of the Zeta sizer (Nano-ZS, Malvern, UK) at 25°C. For the size distribution (polydispersity) study, each sample was diluted appropriately with double distilled water. The data were reported as mean ± SD of triplicate measurements of two independent experiments. The percentage of immunogen entrapment into the liposome was determined: an aliquot of each preparation was centrifuged at 12,000 × *g*, 4°C for 20 min. Amount of the protein in the supernatant was determined (Bradford assay). The percent immunogen entrapment was calculated from the original protein amount added to the liposome.

### Immunization and allergen provocation of the Dp allergic mice

Two weeks after the Dp-CE nebulization (day 41), the Dp-CE allergic mice were divided into five groups of 10 mice each. The mice of groups 1–4 were immunized i.n. with L-CE, L-Der p 1, L-Der p 2, and L-Com, respectively. Mice of group 5 (control) received L-PBS (placebo). All mice were given seven booster doses of the respective vaccines/placebo on alternate days. One week after the last booster (day 62), mice of each treatment group were provoked by nebulizing with aerosolic Dp-CE (10 mg in 10 ml PBS). Sham mice were given eight doses of PBS instead of the vaccines/placebo and received 10 ml PBS nebulization. Two independent experiments were performed.

### Vaccine efficacy evaluation

On day 63 (24 h post-provocation; PP), all mice were bled and their serum samples were tested for CE-specific IgE and IgG1 by indirect ELISA. Thereafter, they were sacrificed as above. Lungs were processed for histopathological study and cytokine gene expressions as described above.

### Statistical analysis

Statistic software SPSS 11.5 was used. Data were analyzed using one-way ANOVA followed by post-hoc comparison using the least significant difference and the independent *t*-test for numbers of inflammatory cells, lung histopathology grades and levels of mRNAs of cytokine genes. Paired *t*-test and the Mann-Whitney U tests were used for analysis of the IgE and IgG1 levels. *P* ≤ 0.05 was considered statistically significant.

## Results

### Dp-CE allergic mice

**Fig 1:**
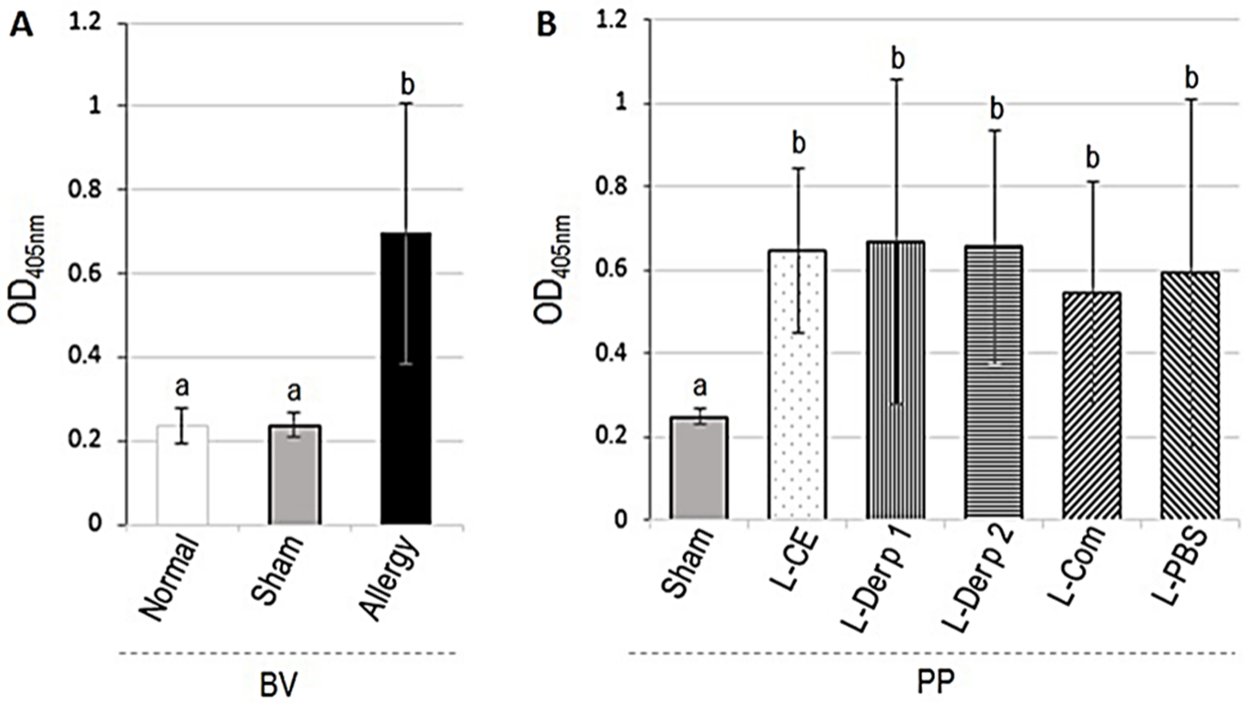
Indirect ELISA OD_405nm_ (means ± SD) of Dp-CE-specific IgE in sera of allergenized mice compared to sham and normal mice (A) before receiving vaccines/placebo (BV). (B) PP, Dp-CE-specific IgE in sera of allergic mice after treatment with vaccines (L-CE, L-Der p 1, L-Der p 2, and L-Com) and provoked with aerosolic Dp-CE compared with placebo (L-PBS) and sham mice. Bars with different superscripts of BV or PP were different at *p* ≤ 0.05.

**Fig 2:**
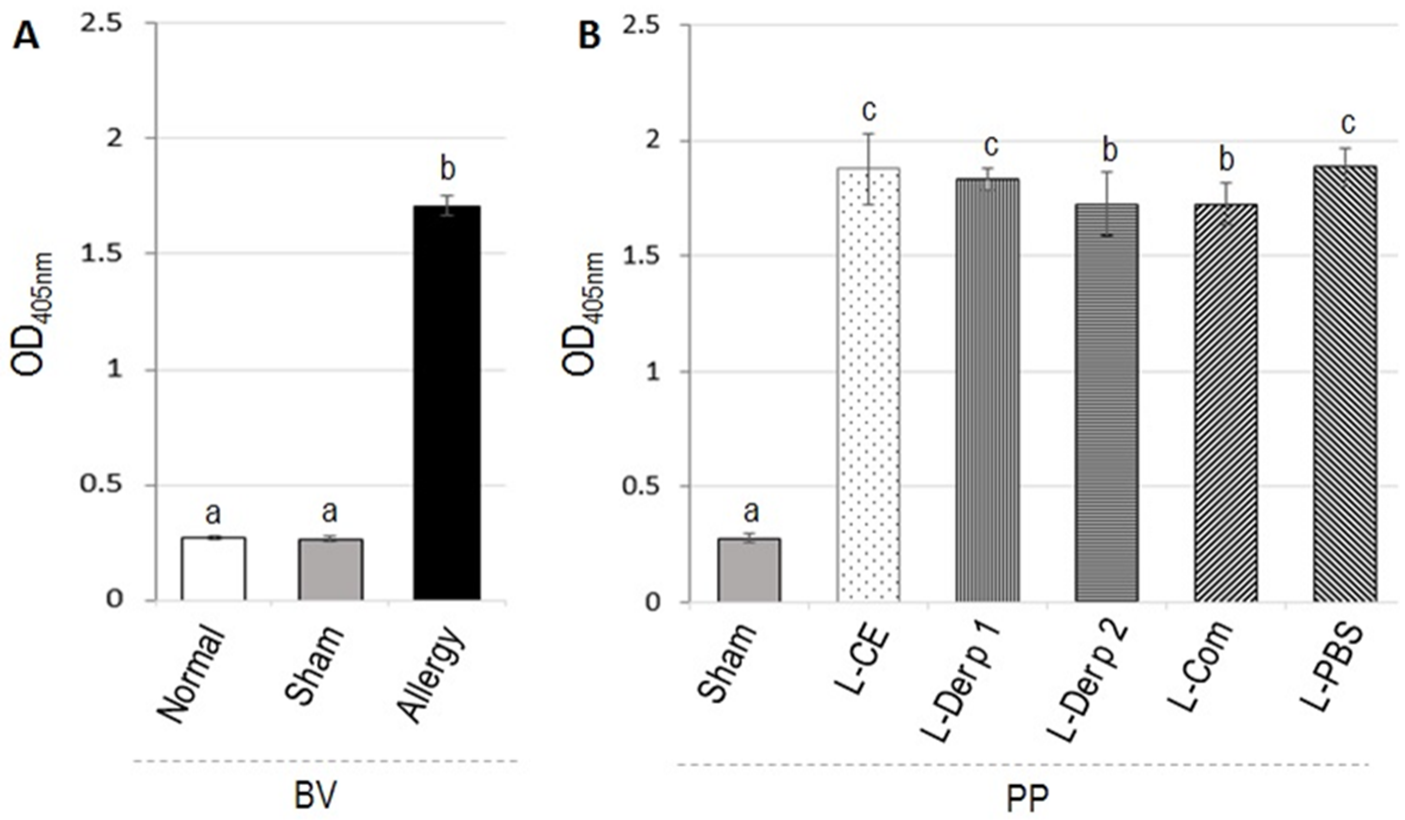
Indirect ELISA OD_405nm_ (means ± SD) of Dp-CE-specific IgG1 in sera of allergenized mice compared to sham and normal mice (A) before receiving vaccines/placebo (BV) and (B) Dp-CE-specific IgG1 in sera of allergic mice after treatment with the vaccines and provoked with aerosolic Dp-CE compared with placebo and sham mice (PP). Bars with different superscripts of BV or PP were different at *p* ≤ 0.05.

The levels of mouse serum specific IgE to Dp-CE determined by indirect ELISA are shown in Fig 1. OD_405nm_ (mean ± SD) of serum IgE of naïve (normal), sham and Dp-CE-allergenized mice were 0.238 ± 0.044, 0.238 ± 0.028, and 0.696 ± 0.314, respectively. The allergenized mice had significantly higher IgE levels than the normal and sham mice (*p* < 0.05) (Fig 1A). Likewise, the allergenized mice had also significantly higher IgG1 level (OD_405nm_ 1.710 ± 0.04) than the normal (0.269 ± 0.01) and sham (0.266 ± 0.01) mice (*p* < 0.05) (Fig 2A). The antibodies of sham and normal mice were not different *(p* > 0.05).

Numbers of inflammatory cells in lungs of the normal, sham and Dp-CE-allergenized mice are compared (Table 1). Average number [mean ± standard deviation (SD)] of the total inflammatory cells (neutrophils, lymphocytes, eosinophils and macrophages) in one microscopic field (magnification 10 × 40) of the lungs of the allergenized mice was 215 ± 91 cells which was significantly higher than those of the sham mice (47 ± 26) and the normal mice (8 ± 7) (*p* < 0.05). Predominant cells infiltrated into the allergenized mouse lungs were lymphocytes and neutrophils while the predominant cells in the sham mouse lungs were neutrophils.

**Table 1:** Inflammatory cells in lungs of Dp-CE allergic mice compared with normal and sham mice before vaccination (BV) and after vaccination/placebo and provocation with Dp-CE (PP).

Group	Mean ± SD of cells/microscopic field*				
	Neutrophil	Lymphocyte	Eosinophil	Macrophage	Total cells
**Before vaccination (BV)**					
Normal	4.46 ± 3.32 ^a^	3.05 ± 6.57^a^	0	0.188 ± 1.68^a^	7.70 ± 7.47^a^
Sham	37.28 ± 25.83^b^	9.275 ± 5.70^b^	0	0	46.55 ± 26.27^b^
Allergenized mice	88.66 ± 43.47 ^c^	125.44 ± 71.89^c^	1.15 ± 2.27 ^a^	0	215.25 ± 90.85^c^
**After vaccination and provocation (PP)**					
Sham	31.76 ± 22.90^b^	11.41 ± 14.01^b^	0	0	43.18 ± 35.02^b^
L-CE	119.33 ± 56.17^c^	61.44 ± 64.87^d^	0.96 ± 1.69^a^	0.10 ± 0.27^a^	181.84 ± 89.86^d^
L- Der p 1	48.73 ± 36.99^d^	37.79 ± 37.75^e^	0.24 ± 0.75^b^	0.05 ± 0.30^a^	86.81 ± 64.31^e^
L-Der p 2	55.42 ± 12.70^e^	36.36 ± 8.47^e^	0.45 ± 0.81^c^	0	92.23 ± 24.47^e^
L-Com	46.31 ± 26.35^d^	35.76 ± 34.16^e^	0.25 ± 0.91^b^	0.05 ± 0.29^a^	82.37 ± 47.74^e^
L-PBS	68.54 ± 44.31^c,e^	41.91 ± 53.89^e^	0.20 ± 1.02^b^	0.04 ± 0.28^a^	110.69 ± 87.30^f^

*, Average of at least 20 microscopic fields (10 × 40) per mouse

Entries with different superscripts (a, b, c, d, e and f) of the same column are statistically different (*p* <0.05)

**Fig 3:**
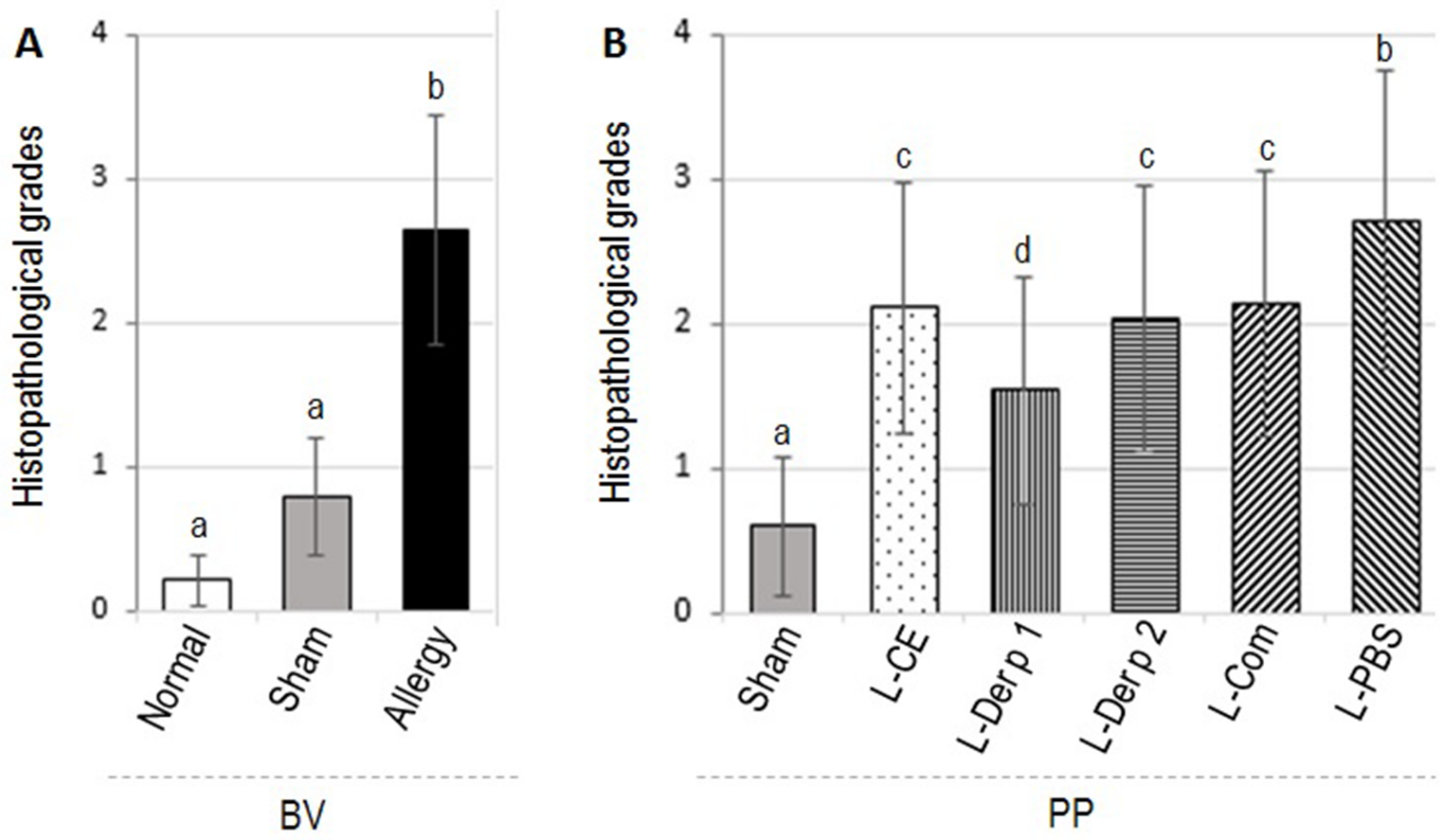
Histopathological grades (mean ± SD) of lung tissues of (A) allergenized, sham and normal mice (BV) and (B) allergenized mice that received vaccines/placebo followed by provocation (PP). Bars with different letters (a and b in A) and (a, b, c, and d in B) are statistically different at *p* ≤ 0.05.

Descriptions for different histopathological grades of mouse lungs are given in S2 Table. Representative appearances of the histopathological grades 0–4 of the mouse lungs are depicted in S1 Fig. Mean ± SD of the histopathological grades of the Dp-CE-allergenized lungs (2.65 ± 0.80) was significantly higher than those of the sham (0.80 ± 0.41) and the normal (0.21 ± 0.42) mice (*p* < 0.05) (Fig 3A).

**Fig 4:**
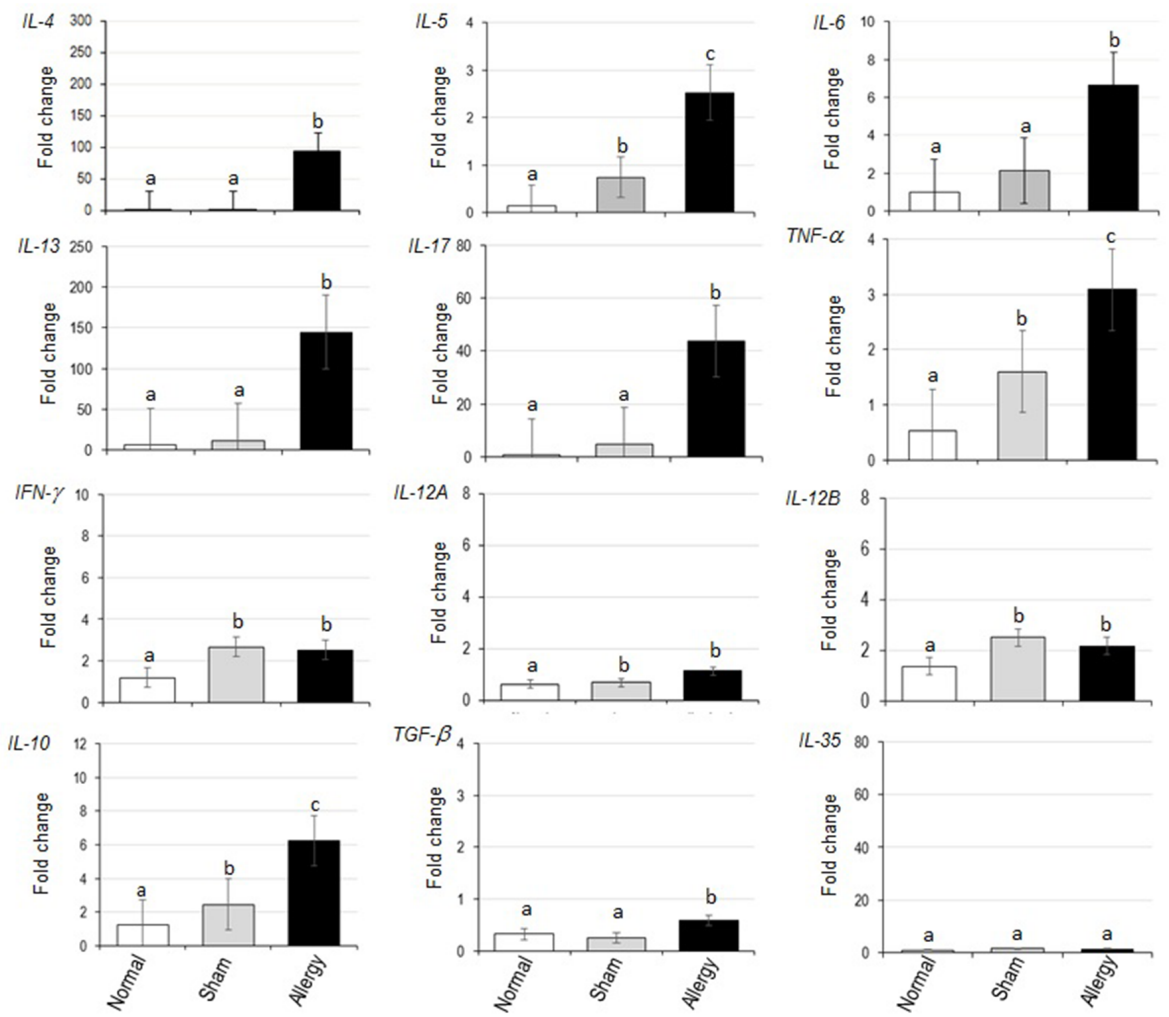
Cytokine expression profiles of normal, sham and allergenized mice.

Fold changes of various cytokine mRNAs in the lungs of normal, sham and allergenized mice compared to β-actin gene mRNA are shown in Fig 4. The Dp-CE-allergenized mice had up-regulations of *IL-4*, *IL-5*, *IL-6*, *IL-10*, *IL-13*, *IL-17*, *TNF-α* and *TGF-β* above the sham and the normal mice. Both sham and allergenized mice had slightly higher expressions of TNF-α, *IFN-γ*, *IL-12A* and *IL-12B* than the normal mice. Based on the serum IgE levels, lung histopathology, infiltrated inflammatory cells, and lung cytokine gene expressions, the Dp-CE-exposed mice were allergic to Dp. They were used further in the efficacy testing of the allergen vaccines.

### Characteristics of liposome and liposome-encapsulated vaccines/placebo

Sizes, polydispersity, zeta potential and % immunogen entrapment of the liposome-encapsulated vaccines and placebo are shown in Table 2. Micelles of all vaccines and placebo were cationic (zeta potentials ranged from 31.33 ± 0.58 to 35.40 ± 0.794) with the sizes ranged from 1.025 ± 0.002 to 4.556 ± 0.023 μm. The mean ± SD of polydispersity indices were 0.311 ± 0.078 to 1.00 ± 0.00. The immunogen entrapments were 81.64 to 90.56%. The L-CE, L-Der p 1, L-Der p 2, L-Com and L-PBS contained 69, 12, 10, 12, and 3 EU of endotoxin/dose, respectively.

**Table 2:** Characteristics of L-CE, L-Der p 1, L- Der p 2, and L-Com vaccines and placebo (L-PBS).

Parameter	Vaccine formulation				Placebo (L-PBS)
	L-CE	L-nDer p 1	L- nDer p 2	L-Com	
Average size (μm) (Mean ± SD)	4.566 ± 0.023^a^	1.999 ± 0.034^b^	2.016 ± 0.120^b^	1.025 ± 0.002^c^	1.93 ± 0.02^b^
Polydispersity (Mean ± SD)	0.311 ± 0.078^a^	0.799 ± 0.071^b^	0.788 ± 0.070 ^b^	0.536 ± 0.020^c^	1.00 ± 0.00^d^
Zeta potential (Mean ± SD)	31.33±0.58^a^	35.40 ± 0.794^a^	33.60 ± 1.058^a^	35.00 ± 0.00^a^	34.3 ± 2.15^a^
% Entrapment	81.64	88.03	90.56	88.34	NA

NA, not applicable

Entries with different superscripts (a, b, c and d) of the same horizontal line are statistically different (*p* <0.05)

Percent entrapment was calculated from one aliquot of each vaccine

### Therapeutic efficacies of the liposome-encapsulated vaccines

Results of indirect ELISA for determining serum specific IgE of the allergic mice after treatment with vaccines/placebo and allergen provocation (PP) are shown in Fig 1B. There was no change in the serum IgE levels in the vaccinated and placebo mice after provocation (PP) compared with the pretreatment levels (BV). Serum IgG1 levels of the allergic mice treated with L-Der p 2 and L-Com were not different from the pretreatment levels after the allergen provocation. IgG1 above the pretreatment levels were found in mice treated with L-CE, L-Der p 1 and placebo (L-PBS) (*p* < 0.05) (Fig 2B).

Histopathological grades (mean ± SD) of lung tissues of allergic mice after treatment with vaccines/placebo and provocation (PP) are shown in Fig 3B. Mice of all vaccinated groups had significant reduction of lung histopathological severity. The histopathology was not changed in the placebo mice. Representative images of the lung histological grades of all groups of allergic mice after treatments are shown in S2 Fig.

Total inflammatory cells in lung tissues of allergic mice that received vaccines/placebo at 24 h post-provocation with aerosolic CE (PP) compared to the cell numbers before treatment (BV) are shown in Table 1. All vaccines were effective in reducing the numbers of the infiltrated cells into lungs of the treated mice. The effectiveness of L-Der p 1, L-Der p 2 and L-Com were not different (*p* > 0.05; superscript e in last column of Table 1). Although the L-CE treated allergic mice had the highest number of infiltrated inflammatory cells in their lungs compared to the other vaccinated mouse groups (superscript d in the last column of Table 1 at PP), but the number was significantly lower than that of the allergic mice at BV (superscript c in the last column of Table 1) (*p* < 0.05). The L-PBS showed some placebo effect on this parameter.

**Fig 5:**
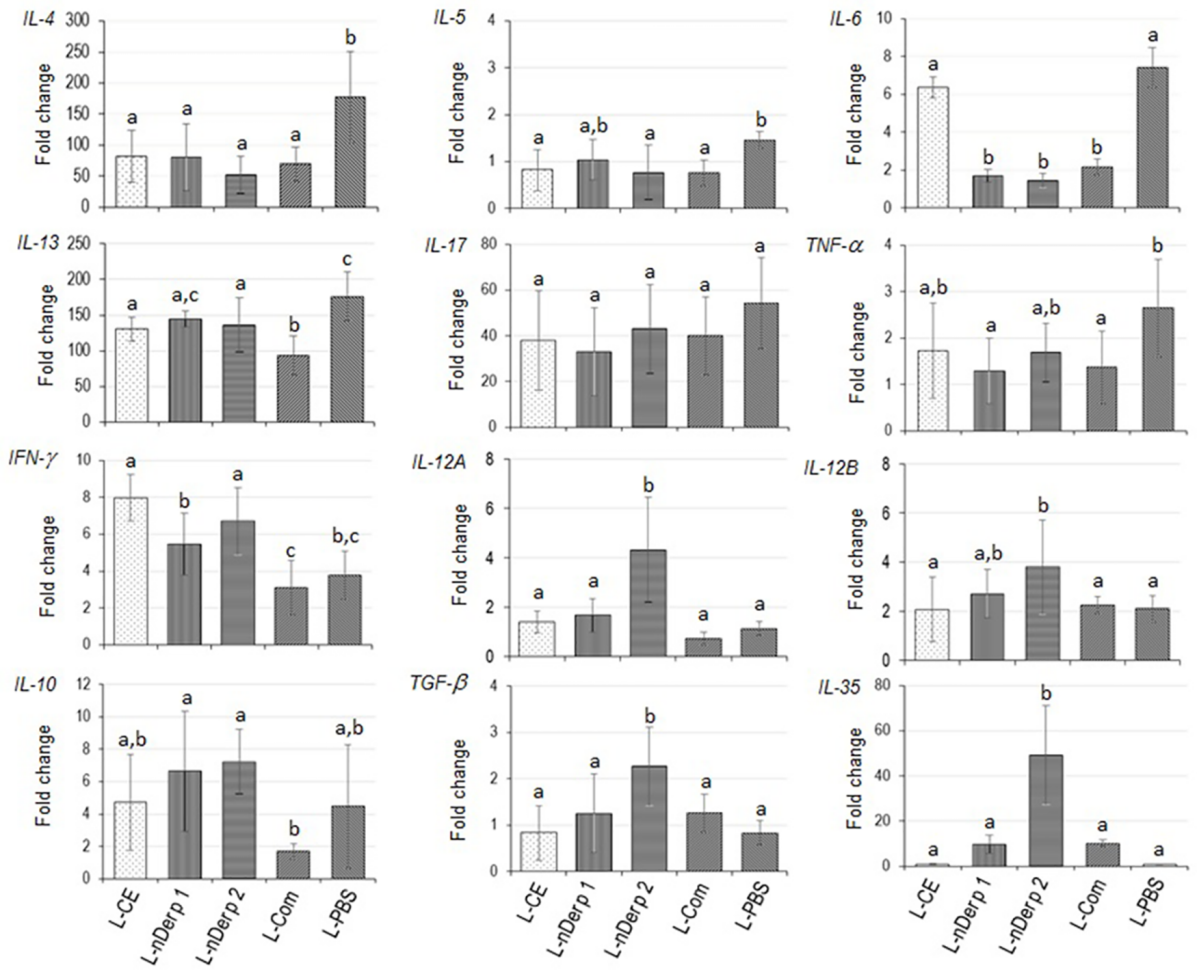
Cytokine expression profiles of *Dermatophagoides pteronyssinus* allergic mice after treatment with liposome-encapsulated vaccines/placebo and allergen provocation.

Fold change of cytokine gene mRNAs in lung tissues of allergic mice after vaccination and provocation in comparison with the β-actin gene expressions are shown in Fig 5. Overall picture of Th2 cytokine expressions of allergic mice treated with all vaccine formulations were lower than the placebo mice (*p* ≤ 0.05). L-Der p 1, L-Der p 2 and L-Com caused also a significant reduction of *IL-6* expression compared to placebo (*p* < 0.05) while the L-CE did not. *IL-17* expression was not different among the treated mouse groups after provocation. L-Der p 2 conferred increases of expressions of *IFN-γ*, *IL-12A* and *IL-12B*, *IL-10*, *TGF-β* and *IL-35 ebi3* compared to placebo (*p* ≤ 0.05). L-Der p 1 caused up-regulation of *IFN-γ* and *IL-10* but not *IL-12*, *TGF-β* and *IL-35 ebi3* compared to the placebo. The L-CE induced significant up-regulation of *IFN-γ* and no different in regulatory cytokine expressions compared to L-PBS. Although the L-Com readily mediated reduction of Th2 cytokine gene expressions (*IL-4*, *IL-5*, *IL13*), *IL-6* and *TNF-α*, this vaccine did not up-regulate expressions of *IFN-γ*, *IL-12*, *IL-10*, *TGF-β* and *IL-35* compared with placebo.

## Discussion

The Dp-CE-allergenized mice were found to have significantly higher allergen-specific serum IgE than the sham and normal mice which conformed to the data reported previously [52]. They also had high IgG1 response. The role of IgG in allergy has been controversial due to their binding affinity to different Fcγ receptors (FcγRs) which may lead to different immune response outcomes, depending on whether the FcγRs are activating (FcγRI and FcγRIIIa) [53] or inhibitory (FcγRIIb) receptors [54,55] on the immune cells [53,56,57]. Thus, the IgG1 that was increased in allergenized mice of this study may have either pathogenic or protective role depending upon the FcγR that they fixed on a particular cell type. The Dp-CE allergenized mice had predominant lymphocyte and neutrophil infiltrations into the lungs with few eosinophils. Although the mouse model of allergy cannot completely resemble human allergic manifestations, it has been reported previously from the data performed on human endobronchial biopsies that there are two distinct pathologic, physiologic and clinical subtypes of asthma [58]. One is eosinophil-positive (a classic eosinophilic process) with associated lymphocytes, mast cells and macrophages and another is eosinophil-negative (nearly absent of eosinophils; a pathologically unclassic process) [58]. In both groups, neutrophils were increased [58]. The increased numbers of neutrophils have been found in a more severe allergic disease [58], in nocturnal asthma [59], and during asthma exacerbation [60]. Previous study [52] reported peaked neutrophil infiltration in bronchoalveolar lavage fluids of the mice at 8 h after the last HDM challenge while the lymphocyte population showed steady increase beyond 8 h to 72 h after last HDM challenge. In another study, OVA sensitized mice had increase in neutrophil numbers from 2 h after intranasal OVA challenge, peaking at 8 h and remained higher than the control mice at 12 and 24 h [61]. In this study, the mice were sacrificed at 24 h post allergen challenge and found high neutrophil number in the allergenized mouse lungs (the earlier time points were not investigated). The Dp-CE mouse model of this study had similar airway inflammatory cell signatures to the previously reported allergic mouse models. Neutrophils were predominant cells infiltrated into the lungs of sham mice which should be due to non-allergy-related tissue irritation and perhaps partly due to endotoxin contained in the PBS (negligible amount) that was used for intranasal challenge (20 μl/mouse) and nebulization (10 ml aerosolic PBS for 10 mice; it is not known how many EU of endotoxin were inhaled by individual mice).

BALB/c mice was chosen for generating the Dp allergy model as they have been shown to develop a vigorous Th2 response due to their inherent robust IL-4 production [62]. BALB/c mice exposed repeatedly to HDM extract exhibited features of airway tissue remodeling including hyperplasia of epithelial cells, metaplasia of goblet cells and subepithelial smooth muscle hyperplasia and hypertrophy which appeared as thickened airway wall [52,63]. In the study, the intraperitoneal route of alum-precipitated Dp-CE was used to prime the allergen-naïve mice as it has been shown that the APCs in the mouse peritoneal cavity were constitutively mature with highly expressed levels of CD40 and B7 co-stimulatory molecules as well as MHC class-II in comparison to the APCs in the respiratory tract which did not express constitutively the maturation markers [64]. After priming, the mice were then challenged via the respiratory route. The average histopathological grade of the allergenized mice was significantly higher than those of the controls, both sham and normal, and also revealed high degree of hypertrophic and proliferative epithelial cells, airway obstruction, and subepithelial smooth muscle hypertrophy, degeneration and detachment from the basement membrane as well as airway wall thickening; all of which are the characteristics of the tissue remodeling. Besides, the allergen-challenged mice had the Th2 response hallmark, i.e., increased expressions of *IL-4*, *IL-5* and *IL-13* and *TNF-α* genes which are the airway cytokine signatures of HDM allergy [52,65]. The Dp-CE allergenized mice had also up-regulations of *IL-17A*, *IL-6*, *IL-10* and *TGF-β*. The IL-17A is a pro-inflammatory cytokine which induced IL-8 causing increased neutrophil influx to the lung; this cytokine also involved in asthma severity, airway remodeling and Treg suppression [66,67]. The IL-6 recruits eosinophils to the lung during the development of allergic airway inflammation; this cytokine causes mucus hypersecretion by airway epithelial cells in response to allergen [68]. IL-6 and TGF-β could induce generation of Th17 cells, termed regulatory Th17 (Treg17) cells that play protective and non-pathogenic role in inflammation while Th17 cells generated through IL-6 and IL-23 induction (termed effector Th17 or Teff17) were pathogenic [69]. Unfortunately, IL-23 expression was not determined in this study. However, high expression of the IL-17 gene indicates that the Teff17 cells were generated after the allergen exposure. Usually IL-10 is a potent anti-inflammatory cytokine which has important role in the regulation of Th2 in allergic response [70–72]. Up-regulation of the *IL-10* in the Dp-CE allergenized mice should reflect an attempt of the immune system to counteract the exacerbating Th2 response. Allergenized mice had up-regulation of *TNF-α*, *IFN-γ* and *IL-12A* and *IL-12B* which should be due to the airway irritation and/or inflammation stimulated by pathogen-associated molecular patterns (PAMPs) such as endotoxin contained in the CE (658.79 EU/ml). The high expressions of *TNF-α*, *IFN-γ* and *IL-12A* and *IL-12B* of sham mice was most likely from tissue irritation as mentioned above. The characteristic features of the Dp-CE-sensitized mice indicated that they were allergic to Dp. Thus the animals were used further for testing of the therapeutic efficacy of the intranasal liposome-encapsulated allergen vaccines.

Various strategies and protocols have been attempted to reduce adverse effects caused by immunotherapy using crude (native) allergenic extracts. These include allergen modifications, altered therapeutic formulations, optimization of the vaccine introduction route, innovative B-cell-focused approach by inducing IgE-competitive IgG response, different choices of vaccine carriers and adjuvants, and combined treatment of the allergy using both AIT and pharmacologic/biologic agent [65]. In this study, we have chosen to use liposome as the vaccine delivery vehicle and adjuvant and the intranasal route of the allergen vaccine administration. It has been shown that the liposome could reduce toxicity/adverse activity of the encapsulated components [73] which in this study were potent Dp allergens. The lipid micelles also protected the content from the hostile environment such as host proteases [74,75]. Liposome acts as an immunological adjuvant for cell-mediated immune response [76]. The phosphatidylcholine and cholesterol used for making liposome of this study are normal constituents of mammalian cells. Thus, the liposome-encapsulated vaccines should be innocuous. It was observed that the allergic mice that received the vaccines did not show any sign of morbidity. All of the formulated Dp vaccines were fairly homogeneous as shown by their polydispersity indices (≤1.0). All vaccines and placebo carried positive charges. Cationic liposome has been shown to coalesce better with the APCs than the anionic counterpart and larger amount of the cargo was delivered from the positively charged liposome directly to the cytoplasm [77] where the antigen processing occurred. Then, the antigenic epitopes in the cytoplasm could be transported to the Golgi apparatus and the endoplasmic reticulum where they can fit to the MHC class-I peptide groove and later expressed on the cell surface for induction of CD8^+^ T cell response [78]. Moreover, large sized-liposome (> 200 nm) that was delivered either parenterally or mucosally had tendency to localize at the early endosome after endocytosis and induced Th1 response to the entrapped antigen [79]. Thus, liposome is well suited as the delivery vehicle and adjuvant of the vaccines that aimed to counteract the pathogenic Th2 response [80]. The intranasal route is non-invasive and relatively immunogen sparing compared to the sublingual immunization. The immune responses induced in the upper airway can be effective at the allergen-induced inflamed lower respiratory tissues due to the common mucosal traffic of the effector cells [81].

Crude mite extracts used in AIT for HDM allergy had several drawbacks. Not only the allergen amounts in the extracts from different lots and sources of the raw materials were varied and difficult to standardize, but also the extracts contained complex mixtures of allergenic and unidentified components. AIT using the crude extract also induced new sensitization to the patients [42]. The well-defined allergenic molecule (exclusion of impurity), particularly the major allergen that is responsible for allergy of most patients has potential advantages over the crude extract as has been advocated [50,82]. The refined allergen is easy to standardize and also free of other unidentified and non-allergenic components. Moreover, the precise immune mechanisms underlying the refined allergen immunotherapy can be investigated without any other confounding factors. Thus in this study, the therapeutic efficacies of vaccines made of refined Der p 1 and Der p 2 or their combination were compared to the crude allergen vaccine as the proof-of-concept.

Allergic mice that received vaccination and placebo did not have any change of serum levels of specific IgE from before vaccination and provocation, indicating that the humoral immune response was already maximal and sustained until the time of vaccine efficacy evaluation. Previous data have shown that the specific IgE levels tended to rise shortly after successful immunotherapy and later reduced [83]. Nevertheless, the reduced levels were not necessarily below the pretreatment levels [83]. Allergic mice vaccinated with the liposome-adjuvanted vaccines and then provoked with the allergen had changes in several other parameters. They had significant reduction of numbers of inflammatory cells and lung histopathology grades compared to pretreatments. Among the four vaccine formulations, the L-CE was the least effective vaccine in decreasing the inflammatory cell infiltration into the lungs which might be due to the relatively high LPS content of this vaccine (69 EU/dose) compared to the other vaccines (12, 10, 12 and 3 EU/dose of L-Der p 1, L-Der p 2, L-Com and L-PBS, respectively) and/or the unidentified allergenic/irritating components contained in the CE that might cause recruitment of the cells. The L-PBS had some placebo effect in terms of reduction of inflammatory cell infiltration into lungs of the treated mice which should be due to the adjuvanticity of the liposome as mentioned previously. Nevertheless, the L-PBS did not confer any reduction in the lung histopathology in the treated mice in contrast to all vaccinated allergic mouse groups that had significant decrease of the lung histopathology.

There were profound changes in the cytokine expression profiles of the vaccinated mice. Although all vaccines caused suppression of Th2 cytokine expressions, only the liposome entrapped single allergen vaccines induced immunosuppressive cytokine gene expressions. L-Der p 1 induced significant up-expression of the *IL-10*. The L-Der p 2 vaccine caused up-regulations of the cytokine genes of Th1 (*IFN-γ* and *IL-12A/B*) and Tregs/Bregs (*IL-10*, *TGF-β* and *IL-35*), indicating that this vaccine could cause immune deviation from the pathogenic Th2 towards the Th1 and the regulatory immune responses. A shift of cytokine profile from Th2 to Th1 (IFN-γ and IL-12) after successful immunotherapy has been observed for AIT of allergies caused by many allergens such as cockroach, grass pollen, insect venom [50,84–86]. Also, it has been known that successful AIT led to induction of Tregs which suppress the effector T cells (Teff) either directly or indirectly (via APCs) rendering anergy of the latter [65,87,88]. The direct suppression of Teff by Tregs can involve several immune-suppressive soluble factors and/or cell-cell contact. Tregs can generate immunosuppressive adenosine or transfer cAMP to the Teff, both CD4^+^ and CD8^+^. Tregs can rapidly suppress TCR-induced Ca^2^+, NF-AT, and NF-κB signaling. Tregs can also produce immunosuppressive cytokines (IL-10, TGF-β, IL-35), and they can suppress by IL-2 consumption or induce Teff death via perforin and granzyme. Furthermore, Tregs can suppress Teff indirectly by down-regulating costimulatory molecules on APCs via CTLA-4 [87,88]. Likewise, the regulatory mechanisms and modulate effects of Bregs have been demonstrated in a variety of chronic inflammations including allergic airway diseases and asthma [89]. Bregs produce IL-10, TGF-β, IL-35, natural IgM, catalytic IgG and/or adenosine and express programmed-death ligand 1 (PD-L1), Fas ligand (FasL), CD38^hi^, CD73^hi^, GITRL, ICAM-1/LFA-1, FcγRIIB, and B cell receptors (BCR) [90]. The Breg-derived IL-10 could control lung inflammation through modulating the T helper balance in mice [91]. Breg TGF-β induced death of Teff, inhibits DC function, enhancing Treg functions and induction of CD4+CD25^hi^Foxp3+ regulatory T cells (converting Teff into Tregs [90–92]. The regulatory function of B cells has been associated with the presence and activation of molecules such as CD40, CD19, CD1d, CD24^hi^ [92,93]. B cells mainly exert their regulatory effect through the inhibition of proliferation and production of pro-inflammatory mediators, such as TNF-*α*, IFN*-*γ, and IL-17 by CD4+ T cells [92]. Bregs also suppress T cell function via IL-10 and PD-L1 [94]. Besides, Bregs have propensity to produce both IgG and IgA that block the harmful factor and impair activity of APCs [89]. IL-35 secreted by both types of regulatory lymphocytes is an anti-inflammatory cytokine that has been known to suppress inflammation [95]. Bregs suppress the function of pathogenic T cells via IL-35, ICAM-1/LFA-1 or FasL [96]. Although the molecular mechanisms of the up-regulated immunosuppressive cytokines that led to mitigation of the tissue inflammation in the vaccinated mice await investigation in detail, the overall results indicate that the regulatory immune response which is the most required immunological features of the allergen-specific immunotherapy could be induced effectively by the liposome-encapsulated refined major allergen vaccines: L-Der p 2 (induced *IL-10*, *TGF-β* and *IL-35* up-regulation) and L-Der p 1 (induced *IL-10* upregulation) and nil by L-CE and L-Com. Inability of a liposome-encapsulated crude allergenic extract in inducing the regulatory response was also observed previously for cockroach allergy [50].

## Conclusions

Mice that showed signatures of allergy to *Dermatophagoides pteronyssinus* (Dp) crude extract were generated. They were treated with eight doses of intranasal liposome-encapsulated Dp vaccines made of refined allergens (L-Der p 1, L-Der p 2 and L-com) in comparison to the vaccine made of crude mite extract (L-CE). All vaccinated allergic mice had significant reduction of lung inflammation and allergen-specific pathogenic Th2 response. Nevertheless, only the liposome-encapsulated single allergens induced immunosuppressive cytokine responses (L-Der p 1 induced expression of *IL-10* and L-Der p 2 induced expressions of *IL-10*, *TGF-β* and *IL-35*). Although allergic mice treated with L-CE caused reduction of airway remodeling and inflammatory cytokine response as well as increased Th1 *IFN-γ* expression, the vaccine caused increased inflammatory cell infiltration into the lungs after allergen provocation [which might be due to the pathogen associated molecular patterns (PAMPs) including endotoxin and/or unknown impurity in the preparation] and failed to generate the regulatory responses which are the most effective immunological features in controlling the allergic diseases. Taken together, the data provide compelling evidence that liposome-encapsulated vaccines made of single refined major allergens were more effective than the vaccine made of crude mite extract in immunotherapy of the mite allergy in the murine model. Clinical efficacy of the L-Der p 1 and L-Der p 2 should be evaluated further.

## Supporting information

S1 Fig Features of lung histologic grades of mice. Panel A, grade 0 (normal lung histology); panels B-E, grades 1–4, respectively.
(PDF)

S2 Fig Representative images of H&E stained lung sections of (A) sham mice (average grade 0.8), (B) allergenic mice treated with L-Der p 1 (averaged grade 1.5); (C) allergic mice treated with L-CE, L-Der p 2 or L-Com (average grade 2.0) and allergic mice treated with L-PBS (placebo) (average grade 2.65) that were correspond to the histologic grades at PP in Fig 3. (PDF)

S1 Table Oligonucleotide primers used for the quantitative real-time PCR (qRT-PCR) in monitoring the cytokine gene expressions. (PDF)

S2 Table Histologic grades and features of lung sections of normal and Dp-CE allergenized mice. (PDF)
