# Supplementary material for: Immunotherapeutic efficacy of liposome-encapsulated refined allergen vaccines against Dermatophagoides pteronyssinus allergy
Source: PLoS One. 2017 Nov 28;12(11):e0188627. doi: 10.1371/journal.pone.0188627 (PMC5705073; doi:10.1371/journal.pone.0188627)

Supporting Information

Efficacy of liposome-encapsulated refined allergen vaccines in immunotherapy of allergy caused by *Dermatophagoides pteronyssinus*

Urai Chaisri[1], Anchalee Tungtrongchitr[2,3], Nitaya Indrawattana[4], Panisara Meechan[3], Watchara Phurttikul[3], Natt Tasaniyananda[3], Nawannaporn Saelim[2,3], Wanpen Chaicumpa[2,3], Nitat Sookrung[3,5,*]

[1] Department of Tropical Pathology, Faculty of Tropical Medicine, Bangkok 10400, Thailand

[2] Department of Parasitology, Faculty of Medicine Siriraj Hospital, Mahidol University, Bangkok 10700, Thailand

[3] Center of Research Excellence on Therapeutic Proteins and Antibody Engineering, Faculty of Medicine Siriraj Hospital, Mahidol University, Bangkok 10700, Thailand

[4] Department of Microbiology and Immunology, Faculty of Tropical Medicine, Bangkok 10400, Thailand

[5] Department of Research and Development, Faculty of Medicine Siriraj Hospital, Mahidol University, Bangkok 10700, Thailand

[*] Corresponding author

E-mail: nitat.soo@mahidol.ac.th (NSR)

S1 Fig. Features of lung histologic grades of mice. Panel A, grade 0 (normal lung histology); panels B-E, grades 1-4, respectively.

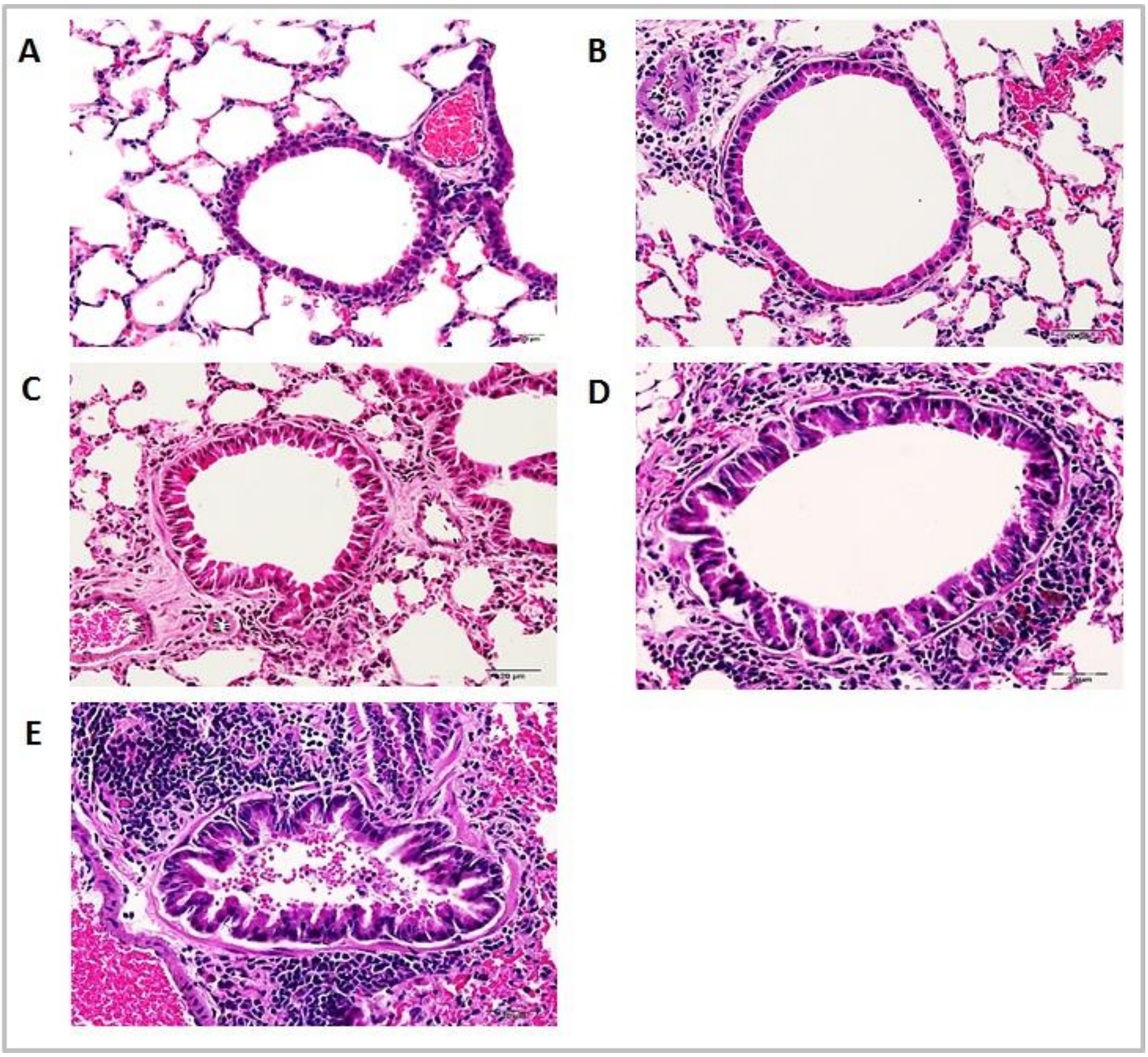

Supplement: S1 Fig — Panel A, grade 0 (normal lung histology); panels B-E, grades 1–4, respectively. (PDF) [file pone.0188627.s001.pdf]
