# Supplementary material for: Immunotherapeutic efficacy of liposome-encapsulated refined allergen vaccines against Dermatophagoides pteronyssinus allergy
Source: PLoS One. 2017 Nov 28;12(11):e0188627. doi: 10.1371/journal.pone.0188627 (PMC5705073; doi:10.1371/journal.pone.0188627)

Supporting Information

Efficacy of liposome-encapsulated refined allergen vaccines in immunotherapy of allergy caused by *Dermatophagoides pteronyssinus*

Urai Chaisri[1], Anchalee Tungtrongchitr[2,3], Nitaya Indrawattana[4], Panisara Meechan[3], Watchara Phurttikul[3], Natt Tasaniyananda[3], Nawannaporn Saelim[2,3], Wanpen Chaicumpa[2,3], Nitat Sookrung[3,5,*]

[1] Department of Tropical Pathology, Faculty of Tropical Medicine, Bangkok 10400, Thailand

[2] Department of Parasitology, Faculty of Medicine Siriraj Hospital, Mahidol University, Bangkok 10700, Thailand

[3] Center of Research Excellence on Therapeutic Proteins and Antibody Engineering, Faculty of Medicine Siriraj Hospital, Mahidol University, Bangkok 10700, Thailand

[4] Department of Microbiology and Immunology, Faculty of Tropical Medicine, Bangkok 10400, Thailand

[5] Department of Research and Development, Faculty of Medicine Siriraj Hospital, Mahidol University, Bangkok 10700, Thailand

[*] Corresponding author

E-mail: nitat.soo@mahidol.ac.th (NSR)

S2 Fig. Representative images of H&E stained lung sections of (A) sham mice (average grade 0.8), (B) allergenic mice treated with L-Der p 1 (averaged grade 1.5); (C) allergic mice treated with L-CE, L-Der p 2 or L-Com (average grade 2.0) and allergic mice treated with L-PBS (placebo) (average grade 2.65) that were correspond to the histologic grades at PP in Fig. 3.

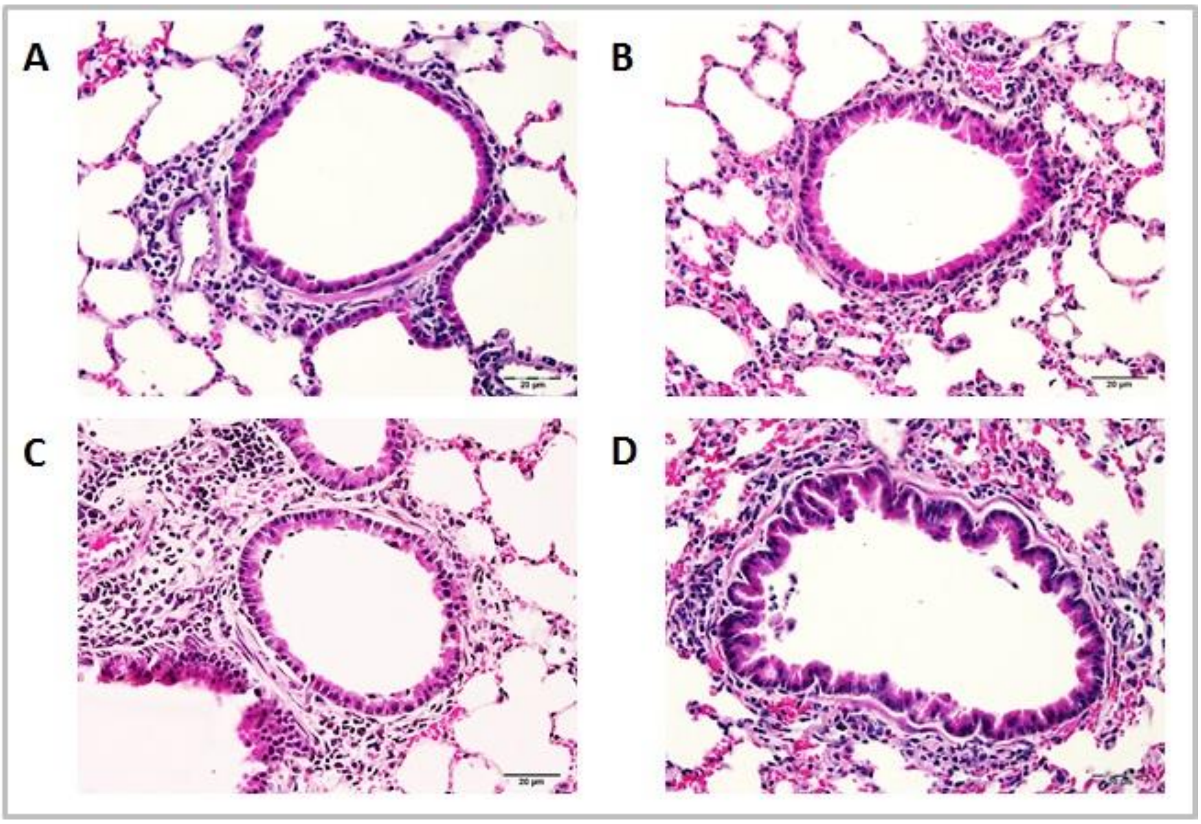

Supplement: S2 Fig — (PDF) [file pone.0188627.s002.pdf]
