# Supplementary material for: Immunotherapeutic efficacy of liposome-encapsulated refined allergen vaccines against Dermatophagoides pteronyssinus allergy
Source: PLoS One. 2017 Nov 28;12(11):e0188627. doi: 10.1371/journal.pone.0188627 (PMC5705073; doi:10.1371/journal.pone.0188627)
Supplement: S1 Table — (PDF) [file pone.0188627.s003.pdf]

Supporting Information

Efficacy of liposome-encapsulated refined allergen vaccines in immunotherapy of allergy caused by *Dermatophagoides pteronyssinus*

Urai Chaisri[1], Anchalee Tungtrongchitr[2,3], Nitaya Indrawattana[4], Panisara Meechan[3], Watchara Phurttikul[3], Natt Tasaniyananda[3], Nawannaporn Saelim[2,3], Wanpen Chaicumpa[2,3], Nitat Sookrung[3,5,*]

[1] Department of Tropical Pathology, Faculty of Tropical Medicine, Bangkok 10400, Thailand

[2] Department of Parasitology, Faculty of Medicine Siriraj Hospital, Mahidol University, Bangkok 10700, Thailand

[3] Center of Research Excellence on Therapeutic Proteins and Antibody Engineering, Faculty of Medicine Siriraj Hospital, Mahidol University, Bangkok 10700, Thailand

[4] Department of Microbiology and Immunology, Faculty of Tropical Medicine, Bangkok 10400, Thailand

[5] Department of Research and Development, Faculty of Medicine Siriraj Hospital, Mahidol University, Bangkok 10700, Thailand

[*] Corresponding author

E-mail: nitat.soo@mahidol.ac.th (NSR)

S1 Table. Oligonucleotide primers used for the quantitative real-time PCR (qRT-PCR) in monitoring the cytokine gene expressions.

Gene	Primer	Size of PCR product (bp)
IL-4	F: 5'-TCGGCATTTTGAACGAGGTC-3'<br>R:5'-GAAAAGCCCGAAAGAGTCTC-3'	218
IL-5	F: 5'-ATGATCGTGCCTCTGTGCCTGGAGC-3'<br>R:5'-CTGTTTTTCCTGGAGTAAACTGGGG-3'	242
IL-6	F: 5'-TCCAGTTGCCTTCTTGGGAC-3'<br>R:5'-GTACTCCAGAAGACCAGAGG-3'	331
IL-13	F: 5'-CGCTGGCGGGTTCTGTGTAG-3'<br>R:5'-GAGGCTGGAGACCGTAGTGGG-3'	121
TNF-α	F: 5'-CATCTTCTCAAAATTCGAGTGACAA-3'<br>R:5'-TGGGAGTAGACAAGGTACAACCC-3'	174
IFN-γ	F: 5'-AACGCTACACACTGCATCTTGG-3'<br>R:5'-GACTTCAAAGAGTCTGAGG-3'	237
IL-12a (p35)	F: 5'- CCACCCTTGCCCTCCTAAAC-3'<br>R:5'- GTTTTTCTCTGGCCGTCTTCA -3'	132
IL-12b (p40)	F: 5'-GGAAGCACGGCAGCAGAATA-3'<br>R:5'-AACTTGAGGGAGAAGTAGGAATGG-3'	180
IL-10	F: 5'-CGGGAAGACAATAACTG-3'<br>R:5'-CATTTCCGATAAGGCTTGG-3'	186
TGF-β	F: 5'-CAAGGGCTACCATGCCAACT-3'<br>R:5'-AGGGCCAGGACCTTGCTG-3'	84
IL-35 (ebi3)	F: 5'-CAATGCCATGCTTCTCGGTAT-3'<br>R:5'-GGACGTGGATCTGGTGGAGTT-3'	84
β-actin	F: 5'-GGCCAACCGTGAAAAGATGA-3'<br>R:5'-CACGCTCGGTCAGGATCTTC-3'	251
