# Supplementary material for: Immunotherapeutic efficacy of liposome-encapsulated refined allergen vaccines against Dermatophagoides pteronyssinus allergy
Source: PLoS One. 2017 Nov 28;12(11):e0188627. doi: 10.1371/journal.pone.0188627 (PMC5705073; doi:10.1371/journal.pone.0188627)
Supplement: S2 Table — (PDF) [file pone.0188627.s004.pdf]

Supporting Information

Efficacy of liposome-encapsulated refined allergen vaccines in immunotherapy of allergy caused by *Dermatophagoides pteronyssinus*

Urai Chaisri[1], Anchalee Tungtrongchitr[2,3], Nitaya Indrawattana[4], Panisara Meechan[3], Watchara Phurttikul[3], Natt Tasaniyananda[3], Nawannaporn Saelim[2,3], Wanpen Chaicumpa[2,3], Nitat Sookrung[3,5,*]

[1] Department of Tropical Pathology, Faculty of Tropical Medicine, Bangkok 10400, Thailand

[2] Department of Parasitology, Faculty of Medicine Siriraj Hospital, Mahidol University, Bangkok 10700, Thailand

[3] Center of Research Excellence on Therapeutic Proteins and Antibody Engineering, Faculty of Medicine Siriraj Hospital, Mahidol University, Bangkok 10700, Thailand

[4] Department of Microbiology and Immunology, Faculty of Tropical Medicine, Bangkok 10400, Thailand

[5] Department of Research and Development, Faculty of Medicine Siriraj Hospital, Mahidol University, Bangkok 10700, Thailand

[*] Corresponding author

E-mail: nitat.soo@mahidol.ac.th (NSR)

S2 Table. Histologic grades and features of lung sections of normal and Dp-CE allergenized mice.

Histologic grade	Histologic features
0	Normal morphology of the bronchiolar epithelium and smooth muscle cells. Single layer of smooth muscle cells at bronchiolar submucosa. The air sacs are clear with delicate lung septa.
1	Bronchiolar epithelium and smooth muscle cells are normal. One layer of inflammatory cells infiltrated into the connective tissue that surrounds the bronchiole.
2	Hyperplasia of bronchiolar epithelial cells and degeneration of smooth muscle cells. Several layers of inflammatory cell infiltration into peribronchiolar areas. Thickened lung septa.
3	Bronchiolar epithelial cells are degenerated and detached from the basement membrane. Marked degeneration of smooth muscle cells that surround the bronchiole. Many layers of inflammatory cells in the peribronchiolar areas. Lung septa are markedly thickened.
4	Sloughing of hypertrophic and proliferated epithelial cells which obstruct the bronchiolar lumen. Necrosis of smooth muscle cells which detached from the basement membrane. Intense inflammatory cells infiltration into the peribronchiolar areas.
